# Liver function test abnormalities and their clinical relevance in primary myelofibrosis

**DOI:** 10.1038/bcj.2017.34

**Published:** 2017-04-21

**Authors:** D Barraco, M Mudireddy, S Shah, C A Hanson, R P Ketterling, N Gangat, A Pardanani, A Tefferi

**Affiliations:** 1 Department of Internal Medicine, Division of Hematology, Mayo Clinic, Rochester, MN, USA; 2 Department of Hematopathology and Cytogenetics, Mayo Clinic, Rochester, MN, USA; 3 Department of Laboratory Medicine and Pathology, Mayo Clinic, Rochester, MN, USA

Hepatomegaly is a common finding in primary myelofibrosis (PMF),^1^ mostly due to extramedullary hematopoiesis, as demonstrated by liver biopsies in previous studies,^2^ and high serum alkaline phosphatase (ALP) is usually the most frequent associated biochemical abnormality. In the current study, we examined the clinical, prognostic and molecular correlates of increased ALP and aspartate aminotransferase (AST) in PMF.

Study patients were selected from our institutional database of myeloproliferative neoplasms and fulfilled the 2016 World Health Organization criteria for the diagnosis of PMF.^3^ Cytogenetic and mutational analyses were performed according to previously described methods.^4^ ALP and AST were chosen as markers of liver function abnormality, based on test availability in the study population. On the basis of preliminary survival analysis and also to be consistent with formal Common Terminology Criteria for Adverse Events (CTCAEs), the optimal cutoff value chosen for abnormally increased ALP was ⩾grade 2 elevation per CTCAE (287 U/l), which is above 2.5-fold increase from the upper limit of the normal range for our institution (45–115 U/l). The optimal cutoff value chosen for AST was ⩾grade 1 elevation per CTCAE (49 U/l; reference range 8–48 U/l). Targeted next-generation sequencing was used to screen for prognostically relevant mutations.^5^ Statistical analyses considered clinical and laboratory data collected at the time of diagnosis or referral to the Mayo Clinic. Differences in the distribution of continuous variables between categories were analyzed by Mann–Whitney test. Patient groups with nominal variables were compared by *χ*2-test. Cox proportional hazard regression model was used for multivariable analysis. *P*-values <0.05 were considered significant. The Stat View (SAS Institute, Cary, NC, USA) statistical package was used for all calculations.

A total of 398 patients with PMF (median age 63 years; 63% males) were considered and their clinical and laboratory characteristics are listed in Table 1. ALP values were available in all patients and AST in 397 cases. ALP was increased to above the upper limit of normal in 190 (48% grade 1 40%, grade 2 7% and grade-3 or above 1%) patients and AST in 34 (9% grade 1 8%, grade 2 one patient). 29 (7%) and 34 (9%) patients displayed the above stipulated increases in ALP (⩾287 U/l) or AST (⩾49 U/l). Dynamic international prognostic scoring system (DIPSS)-plus^6^ risk distributions were 16% low, 17% intermediate-1, 39% intermediate-2 and 28% high. Driver mutation distributions were 64% *JAK2*, 17% *CALR* type 1/type 1-like, 4% *CALR* type 2/type 2-like, 5% *MPL* and 10% triple negative. Cytogenetic studies, available in 390 patients, were abnormal in 36%. Other mutations that were concomitantly analyzed included *ASXL1* (*n*=269), *SF3B1* (*n*=228), *U2AF1* (*n*=257), *SRSF2* (*n*=269) and *TET2* (*n*=92); their respective frequencies were 39, 6, 16, 16 and 16%.

The study cohort was stratified according to the presence of increased ALP ⩾grade 2 (⩾287 U/l, Table 1) and increased AST ⩾grade 1 (⩾49 U/l, data not shown). ALP ⩾287 U/l was associated with marked leukocytosis (leukocyte count >25 × 10^9^/l; *P*=0.0004) and increased AST (*P*=0.04); no other associations were apparent. AST ⩾49 U/l was also associated with leukocytosis as a continuous variable (*P*=0.0003) and leukocyte count >25 × 10^9^/l (*P*=0.003), as well as increased lactate dehydrogenase (*P*<0.0001), increased bilirubin (*P*=0.008), increased ALP (*P*=0.0035), increased alanine transaminase (*P*=0.001) and *SRSF2* mutations (39% mutational frequency vs 13% *P*=0.0012).

After a median follow-up of 3 years, 236 (59%) deaths and 36 (9%) leukemic transformations were documented. In univariate analysis, increased ALP (Figure 1) and AST were associated with inferior survival, both as continuous variables (*P*=0.013 and *P*=0.04, respectively), and categorical variables with the respective cutoff levels of 287 U/l and 49 U/l (*P*=0.03 and *P*=0.03, respectively); the significant difference in survival was independent of DIPSS-plus and of driver mutational status for ALP as continuous (*P*=0.004) and categorical variable (*P*=0.004, hazard ratio 1.84, 95% confidence interval 1.21–2.79), but not for AST (*P*=0.11). Increased serum ALP or AST did not affect leukemia-free survival (*P*=0.4 and *P*=0.5, respectively).

In the current study, we showed that increased level of serum ALP is relatively common in PMF occurring in almost half of the patients at the time of referral, whereas the incidence of elevated serum AST was much less frequent at 9%. The study also shows that the magnitude of the abnormalities were mostly grade 1 for AST, but ⩾grade 2 for ALP in ~7% of the patients. In addition, our study demonstrates significant associations with marked leukocytosis for both ALP and AST. Of note, no other associations were apparent for ALP, but the multiple other associations for AST, which included serum LDH, suggest an indirect effect from the associated leukocytosis. Finally, we demonstrate a DIPSS-plus and driver mutational status independent adverse effect on survival from ALP but not AST. We conclude, therefore, that elevated ALP is frequent in PMF and prognostically relevant when it exceeds 2.5 *χ*, the upper normal limit. Furthermore, the lack of effect on leukemia-free survival suggests that the adverse prognosis associated with ⩾grade 2 increases in serum ALP might be related to excess deaths from disease comorbidity rather than clonal evolution, but additional studies are needed to validate our observations and provide additional insight in the subject matter.

## Figures and Tables

**Figure 1:**
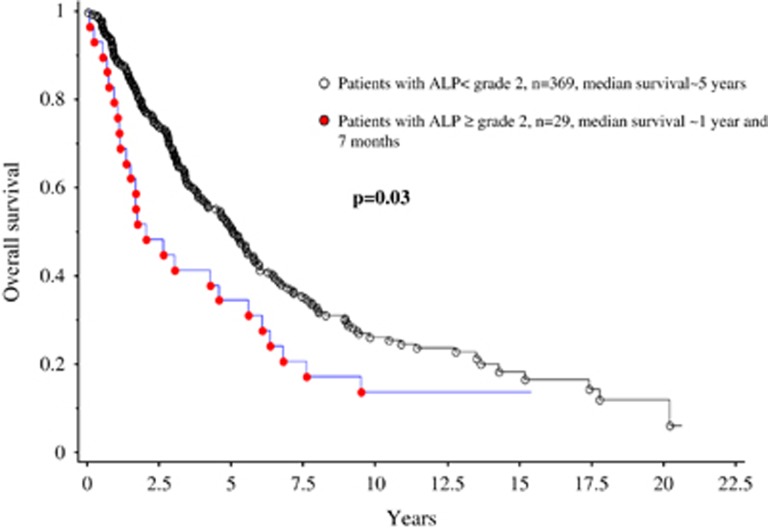
Overall survival in 398 patients with World Health Organization-defined primary myelofibrosis, stratified by the presence or absence of alkaline phosphatase (ALP) ⩾grade 2 elevation per CTCAE.

**Table 1:** Presenting clinical and laboratory of 398 patients with primary myelofibrosis stratified by the presence or absence of alkaline phosphatase ⩾grade 2 elevation per CTCAE

*Variables*	*All patients (*n=*398)*	*Patients with alkaline phosphatase ⩾grade 2 (*n=*29, 7%)*	*Patients with alkaline phosphatase <grade 2 (*n=*369, 93%)*	P*-value*
Age at referall in years median (range)	63 (30–88)	61 (35–81)	62 (28–88)	0.4
Male (%)	249 (63)	21 (72)	228 (62)	0.26
Hemoglobin, g/dl median (range)	10.7 (6.8–16.3)	10.3 (7.4–14.4)	10.7 (6.8–16.3)	0.56
Leukocytes, × 10^9^/l median (range)	8.7 (1.4–146.6)	9.2 (3.7–75)	8.7 (1.4–146.6)	0.06
WBC >25 × 10^9^/l, *n* (%)	52 (13)	10 (34)	42 (11)	**0.0004**
Platelets, × 10^9^/l median (range)	219 (12–1921)	218 (52–662)	219 (12–1921)	0.36
Circulating blasts % median (range)	0 (0–14)	1 (0–10)	0 (0–14)	0.5
LDH, UI/l median (range) ‘*N*' evaluable=346 (87%)	515 (136–2263)	557 (171–1901)	513 (136–2263)	0.71
Total bilirubin, mg/dl median (range) ‘*N*' evaluable=393 (99%)	0.7 (0.1–29)	0.8 (0.2–2.4)	0.7 (0.1–29)	0.5
AST, U/l median (range) ‘*N*' evaluable=397 (99%)	29 (8–145)	33 (14–145)	28 (8–81)	**0.045**
ALT, U/l median (range) ‘*N*' evaluable=96 (24%)	22 (8–209)	35 (12–209)	22 (8–62)	0.22
Presence of palpable splenomegaly ‘*N*' evaluable=396 (99%), *n* (%)	278 (70)	20 (69)	(70)	0.9
Presence of non-hepatosplenic extramedullary hematopoiesis ‘*N*' evaluable=393 (99%), *n* (%)	23 (6)	2 (7)	21 (6)	0.8
Presence of constitutional symptoms ‘*N*' evaluable=392 (98%), *n* (%)	115 (29)	10 (34)	105 (29)	0.5
				
*Bone marrow fibrosis* ‘N'*evaluable=371 (93%)*				
Grade 0 or 1, *n* (%)	47 (13)	3 (11)	44 (13)	0.75
Grade 2 or 3, *n* (%)	324 (87)	25 (89)	299 (87)	
				
*DIPSS-plus*^a^ *risk* ‘N'*evaluable=390 (98%)*				
Low, *n* (%)	62 (16)	6 (21)	56 (16)	0.6
Intermediate-1, *n* (%)	68 (17)	4 (13)	64 (18)	
Intermediate- 2, *n* (%)	151 (39)	9 (31)	142 (39)	
High, *n* (%)	109 (28)	10 (34)	99 (27)	
				
*Driver mutations*				
*JAK2* mutated, *n* (%)	255 (64)	20 (69)	235 (64)	0.7
*CALR* type 1/type 1-like, *n* (%)	67 (17)	6 (21)	61 (17)	
*CALR* type 2/type 2-like, *n* (%)	14 (4)	0 (0)	14 (4)	
*MPL* mutated, *n* (%)	21 (5)	1 (3)	20 (5)	
Triple negative, *n* (%)	41 (10)	2 (7)	39 (11)	
				
*Cytogenetic categories* ‘N'*evaluable=390 (98%)*				
Normal, *n* (%)	248 (64)	19 (66)	229 (63)	0.8
Favorable, *n* (%)	338 (87)	24 (83)	314 (87)	0.52
*ASXL1* mutated ‘*N*' evaluable=269 (68%), *n* (%)	105 (39)	13 (57)	92 (37)	0.07
*SF3B1* mutated ‘*N*' evaluable=228 (57%), *n* (%)	14 (6)	0 (0)	14 (7)	0.2
*U2AF1* mutated ‘*N*' evaluable=257 (65%), *n* (%)	41 (16)	1 (4)	40 (17)	0.1
*SRSF2* mutated ‘*N*' evaluable=269 (68%), *n* (%)	42 (16)	3 (13)	39 (16)	0.7
*TET2* mutated ‘*N*' evaluable=92 (23%), *n* (%)	15 (16)	3 (37)	12 (84)	0.09

Abbreviations: ALT, alanine aminotransferase; AST, aspartate aminotransferase; CTCAE, Common Terminology Criteria for Adverse Events; DIPSS-plus, dynamic international prognostic scoring system-plus; LDH, lactate dehydrogenase; WBC, white blood cell count.

a DIPSS-plus uses eight predictors of inferior survival: age >65 years, hemoglobin <10 g/dL, leukocytes >25 × 10(9)/L, circulating blasts ⩾1%, constitutional symptoms, red cell transfusion dependency, platelet count <100 × 10(9)/L, and unfavorable karyotype (ie, complex karyotype or sole or two abnormalities that include +8, −7/7−, i(17q), inv(3), −5/5q−, 12p−, or 11q23 rearrangement). The presence of 0, 1, ‘2 or 3,' and ⩾4 adverse factors defines low, intermediate-1, intermediate-2, and high-risk disease, respectively. Statistically significant *P*-values are in bold.
